# Seedling growth and fall armyworm feeding preference influenced by dhurrin production in sorghum

**DOI:** 10.1007/s00122-021-04017-4

**Published:** 2022-01-09

**Authors:** Shelby M. Gruss, Manoj Ghaste, Joshua R. Widhalm, Mitchell R. Tuinstra

**Affiliations:** 1 Department of Agronomy, Purdue University, West Lafayette, IN 47907 USA; 2 Department of Horticulture and Landscape Architecture and Center for Plant Biology, Purdue University, West Lafayette, IN 47907 USA; 3 Center for Plant Biology, Purdue University, West Lafayette, IN 47907 USA

## Abstract

Cyanogenic glucosides (CGs) play a key role in host-plant defense to insect feeding; however, the metabolic tradeoffs between synthesis of CGs and plant growth are not well understood. In this study, genetic mutants coupled with nondestructive phenotyping techniques were used to study the impact of the CG dhurrin on fall armyworm [*Spodoptera frugiperda* (J.E. Smith)] (FAW) feeding and plant growth in sorghum [*Sorghum bicolor* (L.) Moench]. A genetic mutation in CYP79A1 gene that disrupts dhurrin biosynthesis was used to develop sets of near-isogenic lines (NILs) with contrasting dhurrin contents in the Tx623 *bmr6* genetic background. The NILs were evaluated for differences in plant growth and FAW feeding damage in replicated greenhouse and field trials. Greenhouse studies showed that dhurrin-free Tx623 *bmr6 cyp79a1* plants grew more quickly than wild-type plants but were more susceptible to insect feeding based on changes in green plant area (GPA), total leaf area, and total dry weight over time. The NILs exhibited similar patterns of growth in field trials with significant differences in leaf area and dry weight of dhurrin-free plants between the infested and non-infested treatments. Taken together, these studies reveal a significant metabolic tradeoff between CG biosynthesis and plant growth in sorghum seedlings. Disruption of dhurrin biosynthesis produces plants with higher growth rates than wild-type plants but these plants have greater susceptibility to FAW feeding.

**Supplementary Information:**

The online version contains supplementary material available at 10.1007/s00122-021-04017-4.

## Introduction

Plants produce many different types of metabolites that can deter insect feeding (Tattersall et al. 2001; Wittstock and Gershenzon 2002) including cyanogenic glucosides (CG). CGs are a family of compounds found in over 2500 plant species (Jones 1998; Conn 1981; Gleadow and Woodrow 2002; Gleadow and Møller 2014), including sorghum, bitter almond (Hosël and Conn 1982), and cassava, and are thought to play key roles in deterring insect feeding (Gleadow and Woodrow 2002). CGs contribute to host-plant resistance to insects by releasing hydrogen cyanide (HCN) when the plant tissues are disrupted by feeding insects (Zagrobelny et al. 2004). HCN inhibits respiration and the utilization of oxygen (Way 1984; Price 1985). HCN also has a bitter taste (Arrázola et al. 2012) that is suspected to deter herbivory leading to the deterrence of feeding in some insects (Gleadow and Møller 2014).

Feeding deterrence can vary in many ways (Gleadow and Woodrow 2002). One of the key factors controlling deterrence is based on the amount of CGs within the plant tissues with higher concentrations leading to greater deterrence, as shown in a study of Japanese beetle feeding in 24 *Prunus* species (Patton et al. 1997). Alternate food sources can also influence feeding patterns as insects will generally avoid feeding on plant tissues with high concentrations of CGs if an alternative food source is available (Gleadow and Møller 2014). Bernays et al. (1977) showed that early instars of *Zonocerua variegatus* would feed on cyanogenic cassava if there were no other choices, but feeding was deterred if another food source was available. The ability of the plant to release HCN is an additional factor that influences insect deterrence. Krothapalli et al. (2013) demonstrated that the $\beta$-glucosidase that catalyzes rapid HCN release in sorghum, dhurrinase2, played a role in insect feeding deterrence, even in the presence of wild-type levels of CG accumulation. Feeding style also influences the role of CGs in host-plant resistance. Tissue disruption is generally required for HCN release. CGs generally have little to no effect on aphids that feed by injecting the stylet through the apoplast of the plant to the phloem, minimizing plant tissue damage (Zhu-Salzman 2004; Gleadow and Møller 2014). The relationship of the insect with the plant can also influence the role of CGs on insect feeding. Opportunistic herbivores may be deterred by cyanogenic plants (Cooper and Swain 1976), while specialists are not deterred because these insects may have evolved tolerance to the CG or have the ability to sequester the CG for defense (Zagrobelny et al. 2004).

Dhurrin is the principle CG produced in sorghum. Sorghum plants contain the highest concentrations of dhurrin during early growth stages and within the youngest plant tissues. Dhurrin concentrations peak within the first few days after germination, and then concentrations decline over time (Busk and Moller 2002; Halkier and Moller 1989). Dhurrin concentrations can increase in older plant tissues in response to environmental factors such as extreme drought, over fertilization, and frost stress (Harms and Tucker 1973; Rosati et al. 2019).

Genetic studies have demonstrated that dhurrin contributes to the deterrence of insect feeding in plants. Tattersall et al. (2001) transformed the biosynthetic pathway for dhurrin into Arabidopsis and showed increased resistance to *Phyllotreta nemorum,* flea beetle. Krothapalli et al. (2013) used chemical mutagenesis to create a knockout of dhurrinase2, the $\beta$-glucosidase that catalyzes the release of HCN from dhurrin and showed increased feeding by fall armyworm (FAW). Other mutations that disrupt dhurrin biosynthesis have also been identified (Blomstedt et al. 2012; Tuinstra et al. 2016). These mutations could increase the safety of sorghum forages by reducing the risk of HCN toxicity, but more work is needed to quantify the impact these mutations have on insect feeding and their implications for primary and secondary metabolism.

Lepidoptera insects and other species that did not evolve as a pest of sorghum are good subjects to study the role of dhurrin in deterrence of feeding (Pentzold et al. 2015). Lepidoptera species have chewing mouthparts that cause the breakdown of dhurrin and release of HCN. FAWs have a wide feeding distribution throughout North and South America (Sparks 1979) and currently are emerging as a significant new pest in Africa (Goergen et al. 2016) and Asia (Chormule et al. 2019), infesting over 80 plant species, including cotton, millet, corn, and sorghum (Day et al. 2017). The distribution and wide range of hosts make FAW a significant pest.

Analyses of host-plant resistance and insect feeding generally rely on destructive methods (Krothapalli et al. 2013; Tattersall et al. 2001) or a ranking system (Diawara et al. 1990) to quantify differences among plant samples. Destructive sampling can be a challenge because each sample provides insight into a single time point, and many samples are required to develop an understanding of changes in feeding preference over time. Alternatively, the ranking scale can be used to assess feeding damage over time, but these measures are subjective and can lead to variation based on the individual ranking the samples. New methods are needed to quickly and accurately phenotype insect feeding characteristics. Nondestructive imaging techniques have shown promise in efforts to classify plant phenotypes in an efficient manner with lower overall costs, time, and labor (Araus and Cairns 2014; Hairmansis et al. 2014). Image-based phenotyping systems have been used to calculate relative growth rate of sorghum based on changes over time (Neilson et al. 2015) and could be beneficial in quantifying feeding damage based on the changes in plant biomass from insect herbivory.

Dhurrin accumulation and turnover play key roles in primary and secondary metabolism by influencing sorghum responses to insect feeding and adaptation to abiotic stresses. In this study, genetic mutants coupled with high-throughput image-based phenotyping techniques were used to explore tradeoffs for manipulating dhurrin metabolism in sorghum under conditions of FAW infestations. Specific objectives included to (1) evaluate the sensitivity of nondestructive plant imaging techniques for quantifying feeding damage in sorghum and to (2) evaluate NILs contrasting for dhurrin accumulation for differences in plant growth and feeding damage following infestation by FAW in greenhouse and field trials. The NILs were selected for variation in *cyp79a1* mutation and the brown midrib (*bmr*) trait. Both traits have value in sorghum when used as a forage. The bmr trait decreases lignin content, thereby increasing digestibility (Porter et al. 1978). CYP79A1 is a cytochrome P450 that catalyzes the conversion of L-tyrosine to *p*-hydroxyphenylacetaldoxime in the biosynthesis of dhurrin (Halkier et al. 1991); thus, the *cyp79a1* trait decreases the risk of HCN toxicity.

## Materials and methods

### Insects

Fall armyworm (FAW) was obtained from Benzon Research (Carlisle, PA) at the 2nd/3rd instar for the calibration experiments and at the 1st instar stage for all subsequent greenhouse and field trials.

### Sorghum genotypes

Three sorghum genotypes were evaluated in the first set of calibration studies: (1) Tx623 (Miller 1978), (2) SbEMS932 *dhr2-1* (mutation in the dhurrinase2 enzyme described by Krothapalli et al. (2013)), and (3) SbEMS2447 *cyp79a1* (C493Y mutation in the CYP79A1 enzyme described by Tuinstra et al. (2016)). The mutants were developed in the Tx623 genetic background but exhibited reduced vigor due to genetic load.

NILs Tx623, Tx623 *bmr6* (Oliver et al. 2005), and Tx623 *bmr6 cyp79a1* were used in all subsequent experiments. Dhurrin contents of Tx623 and Tx623 *bmr6* were determined on seedlings at the third leaf stage through dhurrin extraction and processing through ultra-high performance liquid chromatography (UHPLC) as described below. Dhurrin contents of Tx623 and Tx623 *bmr6* were compared using a *T* test. Tx623 *bmr6 cyp79a1* was developed by crossing and backcrossing (BC1F7) SbEMS2447 *cyp79a1* to Tx623 *bmr6* followed by selfing and selection for no HCN production using the Feigl Anger (FA) assay (Feigl and Anger 1966).

### Imaging platform and calibration

An ARIS TopView Phenotyping System (ARIS, Eindhoven, Netherlands) was used in plant imaging studies. ARIS uses seven channels to calculate the green plant area (GPA), hue, saturation, and other factors. The ARIS system calculates the GPA (mm^2^) based on pixel count.

Calibration studies were conducted using Tx623, SbEMS932 *dhr2-1*, and SbEMS2447 *cyp79a1*. Twenty plants of each genotype were grown to evaluate the use of GPA and changes in GPA over time to quantify insect feeding damage. Sorghum plants were grown in plastic Cone-tainers (Hummert International, St. Louis, MO) that were 120 cm^2^ in a cylindrical shape. The Cone-tainers were filled with the propagation mix (Sun-Gro Horticulture, Agawam, MA) with a single plant per container for approximately 3 weeks. After 2 weeks, the plants were fertilized with Miracle-Gro Garden Feeder (The Scott’s Company, Marysville, OH) at a rate of 350 ppm of nitrogen, 100 ppm of phosphorous, and 200 ppm of potassium. Each plant was tested for repeatability of GPA measurements by first measuring each sorghum plant then measuring them again after 1 h keeping the plant orientation consistent from image to image. This was completed on a whole plant basis. Using the same three genotypes, 16 plants of each were imaged for GPA and total leaf area (mm^2^) of each plant was determined by removing all the leaves and leaf tips at the collar, imaging all the leaves using an RGB camera, and processing imagery with Image J software (https://imagej.net/) for comparison with GPA.

GPA measured by ARIS was also compared with total leaf area (mm^2^) and total dry weight (g) of plants with and without FAW feeding using the same three genotypes described above. Twenty-three plants of SbEMS2447 *cyp79a1* and Tx623 at the 4-leaf stage were placed in one rack, twenty-three plants of SbEMS2447 *cyp79a1* and SbEMS932 *dhr2-1* at the 4-leaf stage were placed in another rack, and sixteen plants of all three genotypes at the 4-leaf stage were placed in the final rack. Each rack was placed into different enclosures and infested with FAW. The enclosures were infested with approximately 150 FAWs at the 2nd instar stage. The FAWs were starved for 2 h before infestation. Infestation occurred for 36 h with ARIS GPA (mm^2^) recorded at 0, 12, 24, and 36 h to track changes across time (Fig. 2) and verify ARIS GPA. After 36 h, each plant was harvested and total leaf area was measured by removing all the leaves and leaf tips at their collar, imaged using an RGB camera, and images processed using Image J software.

This experiment was repeated but with four enclosures: three infested with FAW and one with no insects. Sixteen plants of each genotype were grown to the 4-leaf stage and randomly placed in racks for evaluation with one rack in each of the four enclosures. In addition to imaging comparisons at 36 h, dry weights were determined by drying the plant material in a paper envelope at 60 °C for 1 week.

### Feigl Anger (FA) assay

The NILs Tx623, Tx623 *bmr6*, and Tx623 *bmr6 cyp79a1* were compared for insect susceptibility in greenhouse and the field trials. The phenotypes of plants in each experiment were measured using a FA assay (Feigl and Anger 1966) to verify expected differences in HCN release. The FA assay was conducted by harvesting a 2.5 cm section of the top-collared leaf of a seedling at the 3-leaf stage. The sample was placed in a 96 well plate and frozen in a − 80 °C freezer overnight. The next day the plate was moved to room temperature and the plate was covered with the FA paper and covered with the lid to form a tight seal between the paper and plate. After 15–30 min, the FA paper was removed and photographed to capture differences in blue coloring representing HCN release.

### Dhurrin extraction

The top collared leaf of the plant was weighed for fresh weight and instantly frozen in liquid N. The frozen tissue sample was ground in liquid N. The ground plant samples were extracted using 1:1 MeOH:H_2_O containing 0.05 mg/mL *p*-hydroxybenzaldehyde as an internal standard. The extraction was performed by adding 3 ml of extraction solvent to each 5 mL tube containing ground sample tissue. Tubes were incubated in a water bath at 75 °C for 15 min, then removed, vortexed, and placed at 4 °C for 16–24 h. The next day, the tubes were vortexed and centrifuged and 1 mL of extract was collected and filtered through a 0.2 µm filter into a labeled 1.5 mL microcentrifuge tube. 200 µL of the filtered extract was transferred into vials for ultra-high performance liquid chromatography (UHPLC) analysis.

### UHPLC analysis

UHPLC analysis was performed by using a 1290 Infinity II UHPLC system with a diode array detector (Agilent Technologies, Santa Clara, CA, USA). The separation of compounds was achieved on the Zorbax SB-C18 column (1.8 µm, 2.1 × 50 mm; Agilent) with column temperature maintained at 30 °C. Water (A) and ACN (B) was used as mobile phase solvents with 0.1% formic acid (*v*/*v*) at a flow rate of 0.3 mL min^−1^. The solvent gradient program was set as described in Table 1 with a total run time of 7 min. 5 µL of injection volume was used both for samples and standards.

**Table 1:** UHPLC solvent gradient program

Time (min)	*A* (%)	*B* (%)	Flow (ml/min)
0.00	90.00	10.00	0.300
1.00	90.00	10.00	0.300
3.00	75.00	25.00	0.300
4.00	75.00	25.00	0.300
5.00	5.00	95.00	0.300
6.00	5.00	95.00	0.300
7.00	90	10.00	0.300

**Fig. 1:**
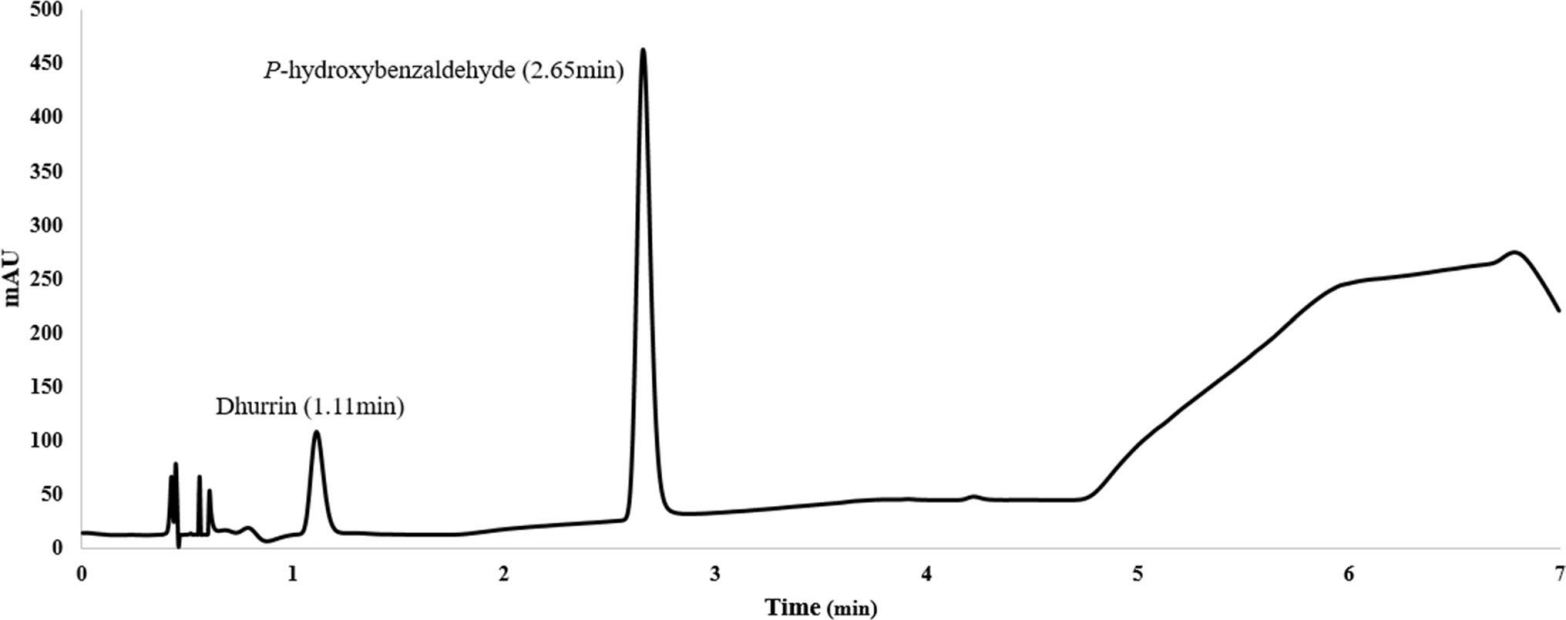
DAD chromatogram (232 nm) of dhurrin (1.11 min) and p-hydroxybenzaldehyde (2.65) at the concentrations of 0.01 and 0.05 mg/mL, respectively

Dhurrin and *p*-hydroxybenzaldehyde were eluted at 1.11 and 2.65 min, respectively (Fig. 1), absorbance for both compounds were recorded at 232 nm. Dhurrin quantitation was performed by analyzing the linear range of 0.0025, 0.0050, 0.0100, 0.0250, 0.0500 mg/mL standards each containing 0.05 mg/mL *p*-hydroxybenzaldehyde as an internal standard. Instrument operation and data analyses were performed using OpenLAB CDS ChemStation software version C.01.09.

### Greenhouse trials

A greenhouse study was designed to compare insect feeding in Tx623, Tx623 *bmr6*, and Tx623 *bmr6 cyp79a1*. Two NILs differ for the *bmr6* trait, and two NILs differ for the *cyp79a1* trait allowing tests of both traits on insect susceptibility. The experiment was conducted using a randomized complete block design with each genotype represented by 16 plants in each of four enclosures using plants of similar size at the 3-leaf stage. One enclosure was not infested with FAW and the other three insect treated enclosures were infested with approximately 150 FAWs at the 1st instar stage to avoid any increased tolerance in the insects due to age. The duration of feeding was increased to 96 h compared to the 36 h in the calibration study to provide an opportunity for significant insect damage to occur in each enclosure with ARIS GPA taken every 24 h. After 96 h, dry weight (g) of each plant was determined as described in the calibration studies.

### Field trials

Field trials were conducted in 2019 and 2020 using the NILs Tx623, Tx623 *bmr6*, and Tx623 *bmr6 cyp79a1*. The plots were overplanted and thinned to 85,000 plants ha^−1^ in 0.76 × 3.1 m plots to plants of similar size and leaf stage with five replications in 2019 and six replications in 2020 in a split plot design with FAW infestation as whole plots and genotype as subplots. Each subplot was surrounded by a barrier using lawn edging (Master Mark, Paynesville, MN) to limit FAW movement within the subplots. Two rounds of this experiment were completed in the summer of 2019 in June and July, and the third round was completed in June 2020. All three experiments were planted at the Purdue Agronomy Center for Research and Education. In 2019, plants were infested with FAW at the 3- to 4-leaf stage using a bazooka applicator (Wiseman and Widstrom, 1980) with 4–5 insects per plant at the 1st instar stage for round 1 and 6–7 insects per plant for round 2. Two replications were harvested at 5 days and three replications harvested at 10 days. Twenty plants per plot were measured for dry weight (g). A subsample of four plants was measured for total leaf area (cm^2^) using a LI-3100C Leaf Area Meter (LI-COR Biosciences, Lincoln, NE). A similar experiment was conducted in 2020 with six replications. Plants at the 3-leaf to 4-leaf stage were treated with FAW at the 1st instar stage on June 23, 2020 using a bazooka applicator at a rate of 4–5 insects per plants. Plots were harvested on day 12 with a longer infestation time due to cool weather and slow feeding rates. Ten plants were harvested per plot and measured for total dry weight (g) and four plants were measured for total leaf area (cm^2^).

### Statistical analysis

Regression analyses were used to compare GPA to total leaf area (mm^2^) and dry weight (g) over time. Outliers for each dataset was detected using boxplots. The boxplots were designed to describe the dependent variable; green plant area, dry weight, or total leaf area, based on the entry, treatments, and hours if captured. Outliers were removed from the dataset (Supplementary information, Fig. S1 and Fig. S2).

A mixed effects model was used to determine the effects of the insect treatments on the different genotypes in the greenhouse experiments. Entry, Treatment, Hours, and their interactions were treated as fixed effects and replications nested within hours were treated as random effects. Least square means were compared using Tukey’s test with Kenward–Rogers degrees of freedom.

A mixed effects model was also used to determine the effects of the insect treatments on the different genotypes in the field experiments. Fixed effects were entry, treatment, days, and interactions for 2019, while days were not a factor in 2020. Replication was evaluated as a random effect. Each experiment was analyzed separately and in a combined analysis. Least square means were compared using Tukey’s test with Kenward–Rogers degrees of freedom. Dry weights were transformed with a square root transformation to fit a normal distribution. The transformed data were used to screen for outliers, build the mixed effects model, and for Tukey’s test.

## Results

### Nondestructive plant imaging to quantify insect feeding damage

**Fig. 2:**
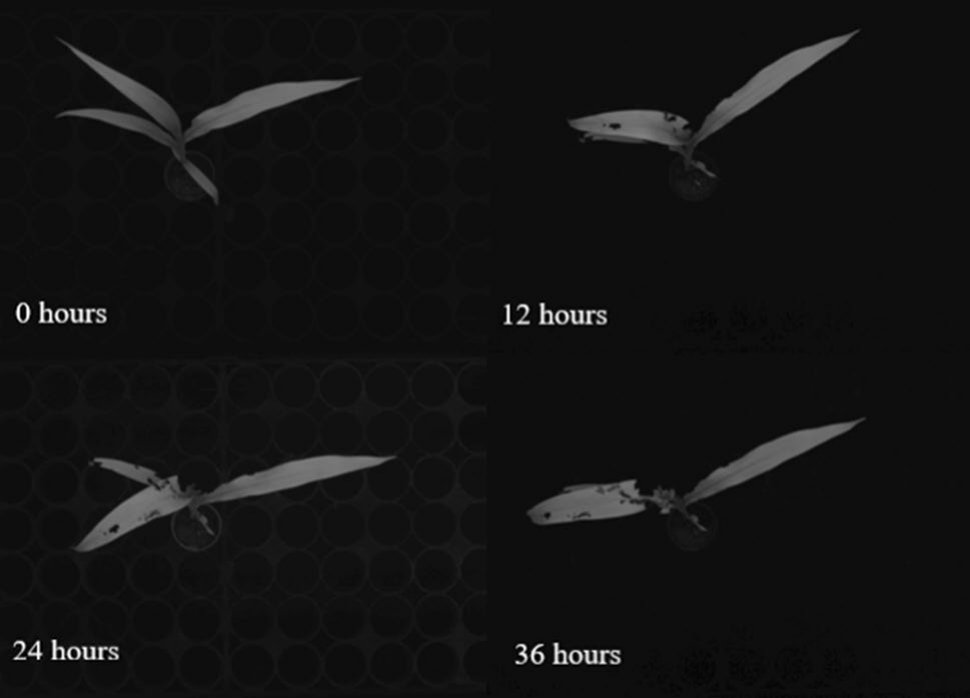
Infested sorghum plant imaged at 0, 12, 24, and 36 h after FAW infestation

**Fig. 3:**
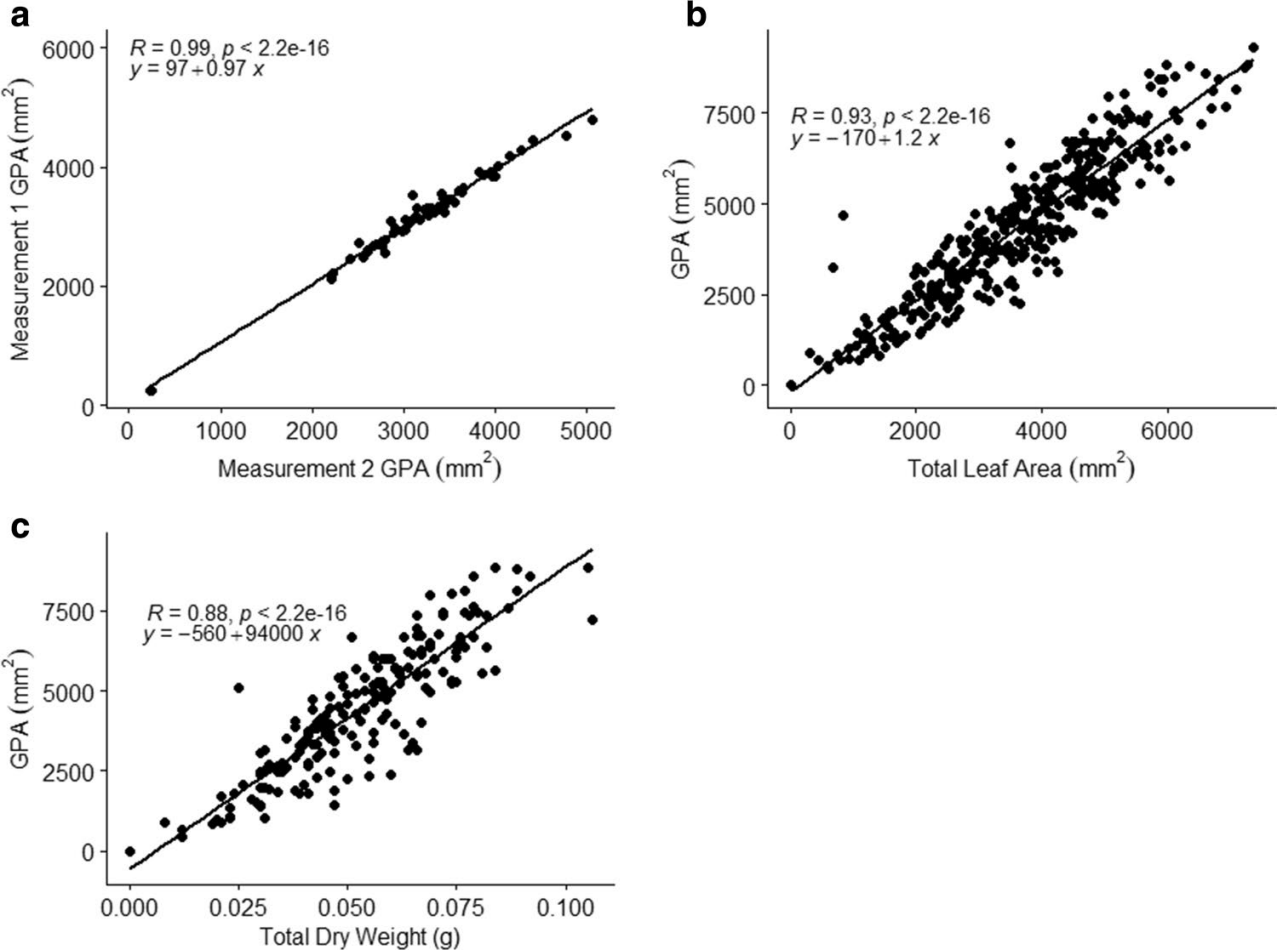
Linear regression models comparing **a** consistency of GPA across measurements, **b** GPA (mm^2^) to total leaf area (mm^2^) from processing with ImageJ, and **c** GPA (mm^2^) to total dry weight (g)

Calibration studies were conducted to determine the repeatability of green plant area (GPA) measurements from the ARIS TopView Phenotyping System. Figure 2 shows the mask created to calculate GPA across time and insect feeding. The average GPA measurements for the first set of measurements was 3155.6 mm^2^, and the second set of measurements average was 3165.2 mm^2^. Repeated measures were compared by regression with an *R*^2^ of 0.98 (Fig. 3a). Comparisons of GPA with total leaf area measured by destructive sampling and fall armyworm (FAW) feeding showed an *R*^2^ of 0.86 (Fig. 3b). Comparisons of the GPA with total dry weight showed similar results with an *R*^2^ of 0.77. Pearson’s Correlation showed a correlation of 0.93 compared to total leaf area and a correlation of 0.88 with dry weight (Fig. 3c). The correlation between the GPA and the plant fresh weight was not as high with an *R*^2^ of 0.61 (not shown).

### Dhurrin production and HCN release

**Fig. 4:**
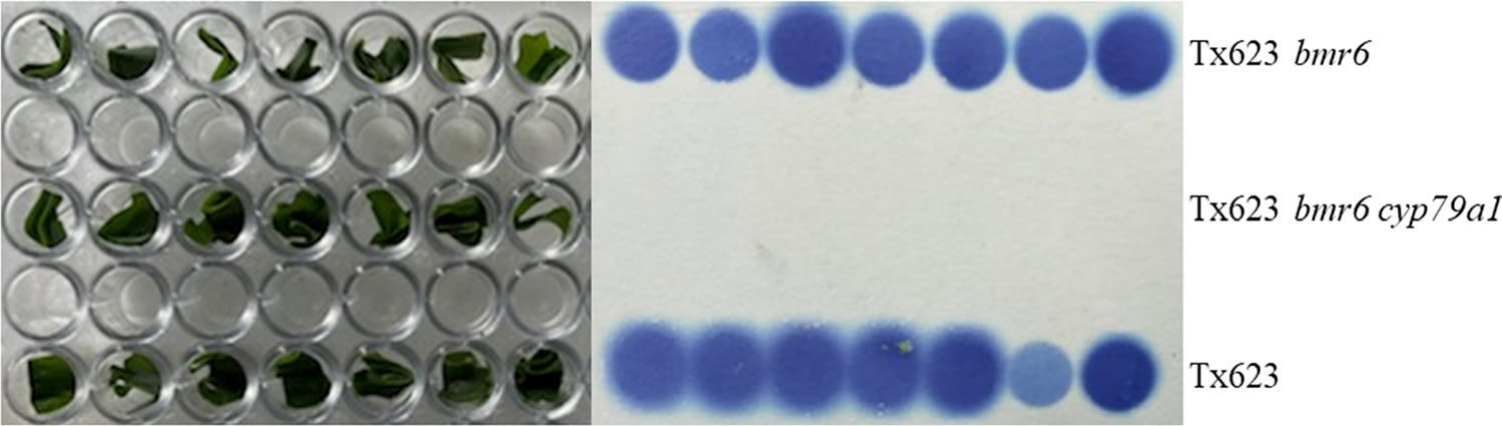
Feigl Anger (FA) assay comparing the NILs for HCN (blue) or no HCN (white) production

Analyses of dhurrin content of the NILs Tx623 (5.2 mg g^−1^) and Tx623 *bmr6* (6.25 mg g^−1^) at the 3-leaf stage indicated no significant differences among lines. Analyses of HCN release before each experiment showed that Tx623 and Tx623 *bmr6* released HCN within 15 min after thawing (Fig. 4). Tx623 *bmr6 cyp79a1* did not show any release of HCN by remaining white (Fig. 4).

### Dhurrin biosynthesis influences sorghum growth and fall armyworm feeding preference

**Fig. 5:**
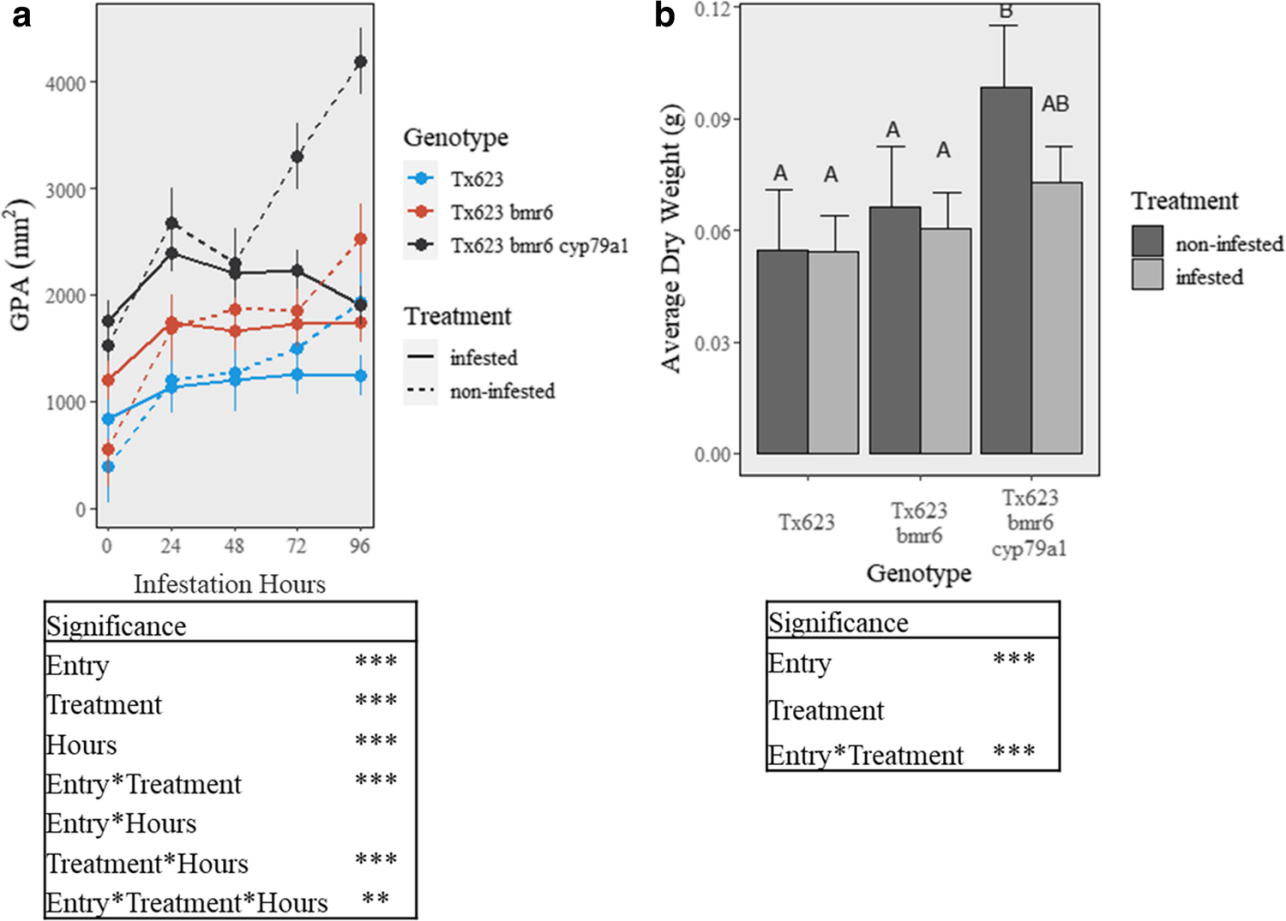
Effects of the *cyp79a1* and *bmr6* mutations on FAW feeding in greenhouse trials of sorghum NILs contrasting in dhurrin production based on **a** changes in GPA over time after infestation and **b** average dry weight at 96 h with the letters representing Tukey’s comparisons across genotypes and treatments. **α = 0.01, ***α = 0.001

NILs of Tx623 contrasting for *cyp79a1* and *bmr6* mutations were compared for plant growth and susceptibility to FAW feeding under infested and non-infested conditions in greenhouse trials. Under non-infested conditions, the GPA for each of the three NILs increased over time as the plants maintained rapid growth (Fig. 5a). The dhurrin-free Tx623 *bmr6 cyp79a1* plants exhibited significantly higher GPA (Fig. 5a) and dry weight (Fig. 5b) than Tx623 and Tx623 *bmr6* plants at 96 h under non-infested conditions. Conversely, GPA remained steady or decreased over time in the sorghum NILs infested with FAW (Fig. 5a). The GPA of the infested Tx623 *bmr6 cyp79a1* plants decreased over time as the feeding trial progressed (Fig. 5a). Further analyses of GPA indicated that the insects began to devour enough plant material to measure a difference in plant area for Tx623 *bmr6 cyp79a1* between 48 and 72 h while Tx623 and Tx623 *bmr6* did not exhibit a difference until 72 and 96 h (Fig. 5a). The Tx623 *bmr6 cyp79a1* plants exhibited the largest difference in GPA (Fig. 5a) and dry weight (Fig. 5b) between the infested and non-infested treatments at 96 h demonstrating feeding preference for dhurrin-free plants.

The sorghum NILs were also evaluated for variation in leaf area and dry weight under FAW infested and non-infested conditions in field trials conducted in 2019 and 2020. The FAW treatment effects were significant in each study with infested plants exhibiting similar or lower total leaf areas and dry weights than non-infested plants for each genotype (Table 2). Genotype by treatment interaction effects was observed for total leaf area in one study in 2019 and for dry weight in both trials conducted in 2019 (Table 2). Tx623 *bmr6 cyp79a1* was the only NIL in the study that exhibited significant differences in total leaf area and dry weight between infested and non-infested treatments in all three studies (Table 2). These differences demonstrate that more FAW feeding occurred in the dhurrin-free Tx623 *bmr6 cyp79a1* as compared to Tx623 and Tx623 *bmr6*. Although more susceptible to FAW feeding, the dhurrin-free plants did not exhibit differences in leaf area or dry weight from the wild-type plants in the FAW infested treatments due to increased size of the dhurrin-free plants prior to infestation.

**Table 2:** Variation in leaf area and dry weight of sorghum NILs contrasting for brown midrib (*bmr6*) and dhurrin biosynthesis (*cyp79a1*) mutations under fall army worm (FAW) infested and non-infested conditions in field trials in 2019 and 2020

Entry	FAW treatment	Total leaf area (cm^2^)					
		2019–1^a^		2019–2		2020	
Tx623 *bmr6 cyp79a1*	No	251	a	568	a	1069	a
Tx623 *bmr6*		108	b	501	ab	995	ab
Tx623		241	ab	467	ab	973	abc
Tx623 *bmr6 cyp79a1*	Yes	149	b	366	bc	887	bc
Tx623 *bmr6*		90	b	368	bc	834	bc
Tx623		188	ab	543	ab	785	c
Significance^b^	Entry	***					
	Treatment	**		*		***	
	Entry * Treatment			**			

Entry	FAW treatment	Total dry weight (g plant^−1^)					
		2019–1		2019–2		2020	
Tx623 *bmr6 cyp79a1*	No	1.57^c^	a	3.26	a	9.93	a
Tx623 *bmr6*		0.84	b	2.59	b	9.93	ab
Tx623		1.58	a	2.6	b	9.12	abc
Tx623 *bmr6 cyp79a1*	Yes	0.99	bc	2.35	bc	8.31	c
Tx623 *bmr6*		0.6	b	2.18	bc	8.7	abc
Tx623		1.12	bc	3.02	ab	9.34	abc
Significance	Entry	***		**			
	Treatment	***		**		***	
	Entry * Treatment			***			

Differences between values are shown using Tukey’s Test. Analyses of variance tests were used to test significance of Entry, Treatment, and Entry x Treatment interactions

^a^For the 2019 data, the day 5 values are not presented because there were no significant effects

^b^*0.05, **0.01, *** = *0.001*

^c^All average values are based on the original data

## Discussion

Many studies have demonstrated the value of image-based sorghum phenotyping technologies for assessment of plant growth and development (Neilson et al. 2015; Batz et al. 2016; Thapa et al. 2018; Masjedi et al. 2020). In this study, the ARIS phenotyping platform was shown to be useful for collecting nondestructive measurements of green plant area (GPA) in sorghum. Estimates of GPA were consistent over time and compared favorably with estimates of total leaf area calculated using ImageJ software and total dry weight for insect feeding damage. Unlike destructive measurements of insect feeding based on changes in dry weight and leaf area, the ARIS platform was useful for taking nondestructive measurements of the same plant over a time when the insects were feeding. The platform was able to detect a decrease in leaf area compared to the non-infested plants due to feeding within and along the leaf margins. This image-based technique also avoids challenges with subjective ratings, provides for measurements at multiple time points, and is quick and easy to use. The opportunity for taking multiple readings per plant was particularly useful for measuring plant–insect interactions among genotypes. This provided unique insights into the feeding patterns of the insects throughout the infestation period.

Fall armyworm (FAW) generally is a pest of sorghum at later stages of development when the whorl is developed or the paniclse of sorghum plants have emerged (Diawara et al. 1990). In this study, sorghum plants were infested at earlier stages of development to study FAW feeding preference when dhurrin content is generally higher than observed at later stages of development (Burke et al. 2013). Dhurrin content can vary depending on environmental stresses including drought (O’Donnell et al. 2013; Rosati et al. 2019), frost (Gleadow and Møller 2014; Strickland et al. 2017), and over-fertilization (Busk and Moller 2002). Wiseman and Gourley (1982) showed that seedling plants have consistent patterns of resistance at the whorl stage and can be used to study patterns of susceptibility and resistance to FAW feeding. Comparisons of infested and non-infested plants demonstrated significant differences in GPA and dry weight with infested plants consistently smaller than the non-infested plants in each round of the experiment. Interactions between genotypes and infestation treatments provided evidence for differences in susceptibility to FAW feeding. FAW feeding studies in greenhouse trials produced plant damage more quickly than in field studies. This could be due to a more consistent and favorable environment for plant and insect development within the greenhouse. Rain, wind, cool nights, and predators such as birds may have slowed FAW feeding damage in the field studies.

NILs contrasting for *bmr6* responded to the FAW feeding in field and greenhouse trials with a pattern of response to FAW infestation similar to studies described by Dowd and Sattler (2015). The wild-type and *bmr6* plants had similar dry weights in the infested and non-infested treatments at 96 h. These observations suggest that the plants were growing at a similar rate and with similar FAW feeding over time. NILs contrasting for *cyp79a1* exhibited a different pattern of plant growth and feeding preference. The *cyp79a1* plants generally produced higher GPA and dry weights under non-infested conditions compared to wild-type plants in greenhouse and field trials. This observation suggests that carbon and nitrogen pools otherwise committed to dhurrin biosynthesis may be redirected to support a higher growth rate of vegetative tissues. This differs from what was reported for the totally cyanide deficient mutant1 (*tcd1*), that exhibited delayed germination (Montini et al. 2020), reduced growth during early seedling growth (Blomstedt et al. 2012), and altered flowering times (Sohail et al. 2020). The reduced growth during germination in *tcd1* plants likely highlights importance of recycling dhurrin for germination and early seedling growth (Montini et al. 2020).

While *cyp79a1* and *tcd1* both lead to mutations in CYP79A1 and loss of dhurrin production, they lead to changes at different residues in the enzyme. The *cyp79a1* mutant contains a C493Y mutation (Tuinstra et al. 2016), while *tcd1* bears a P414L mutation (Blomstedt et al. 2012). CYP79A1 catalyzes two N-hydroxylation reactions to afford a highly unstable *N*,*N*-dihydroxytyrosine intermediate, which is subsequently dehydrated and decarboxylated to form the final CYP79A1 product, (E)-p-hydroxyphenylacetaldoxime (reviewed in Sørensen et al. 2018). It therefore seems reasonable to predict that the C493Y and P414L mutations may have different structural consequences that could lead to the accumulation of different CYP79A1 reaction intermediates that might also contribute to the different phenotypic differences observed in the dhurrin-free *tcd1* and *cyp79a1* mutants.

In contrast to non-infested conditions, the growth of *cyp79a1* plants was reduced compared to wild-type plants upon infestation by FAW. The infested *cyp79a1* plants were consistently smaller than the non-infested plants and showed the largest reduction in GPA and dry weight over time following infestation by FAW. This indicated that the wild-type plants were more resistant to insect feeding than dhurrin-free plants. This is consistent with reports of increased insect resistance when introducing dhurrin biosynthesis into Arabidopsis (Tattersall et al. 2001) and greater susceptibility in sorghum plants having reduced capacity to release HCN due to a genetic mutation in *dhurrinase2* (Krothapalli et al. 2013). In both cases, the insect species had chewing mouthparts that are necessary to release HCN during feeding.

Manipulation of dhurrin metabolism may provide a strategy to increase sorghum growth rate when infestations of insects such as FAW are controlled; however, dhurrin metabolism is complex and reported to play a role in nitrogen use efficiency (Neilson et al. 2015; Blomstedt et al. 2018; Rosati et al. 2019) and drought tolerance (Burke et al. 2013; Adeyanju et al. 2016; Hayes et al. 2016; Varoquaux et al. 2019). Follow-up studies are needed to examine the metabolic tradeoffs that influence sorghum germination, productivity, and sustainability with variable nitrogen and water inputs.

## Conclusions

Dhurrin metabolism plays an important role in sorghum resistance to FAW feeding. Given the tremendous flux of carbon and nitrogen through this metabolic hub, dhurrin production also influences plant growth and development. This study demonstrated that a genetic mutation that disrupts dhurrin biosynthesis also positively impacts plant growth and development in non-infested growing conditions. Taken together, these studies revealed a significant metabolic tradeoff between CG biosynthesis and plant growth in sorghum seedlings. These results suggest that it may be possible to manipulate dhurrin metabolism to optimize sorghum productivity in diverse production environments.

## Supplementary Information

Below is the link to the electronic supplementary material.

Figure S1. Boxplots showing distributions of the greenhouse data and outliers for (a) ARIS GPA and (b) dry weight. (PPTX 289 KB)

Figure S2. Boxplots showing the distributions of the field data and outliers for (a-c) dry weight and (d-f) total leaf area. (PPTX 314 KB)

## Data Availability

Plant growth and development data from the greenhouse and field trials described in this publication are available at the Purdue University Research Repository (PURR) (Gruss and Tuinstra, 2021). The files are designated by location and year with locations shown as greenhouse (GH) or field (WL) then the year the study took place. For example, the GH18_insect_data.xlsx includes data from the greenhouse trials in 2018. Within each file, there may be multiple sheets that define the type of data collected. Data for green plant area collected with the Aris TopView Phenotyping System, total leaf area, fresh weight, and dry weight are organized by plot, genotype, and insect infestation treatment. Temporal data from the greenhouse trials include time (hours) when measurements was taken, the plant number, and insect infestation treatment. More details of each of the data and R code files are included in the READ_ME file (Gruss and Tuinstra, 2021).

## Material availability

Seeds of genetic mutants are available for non-commercial research purposes under a material transfer agreement with Purdue University.
