# Supplementary figures and images for: Seedling growth and fall armyworm feeding preference influenced by dhurrin production in sorghum
Source: Theor Appl Genet. 2022 Jan 9;135(3):1037–47. doi: 10.1007/s00122-021-04017-4 (PMC8942933; doi:10.1007/s00122-021-04017-4)

## Slide 1
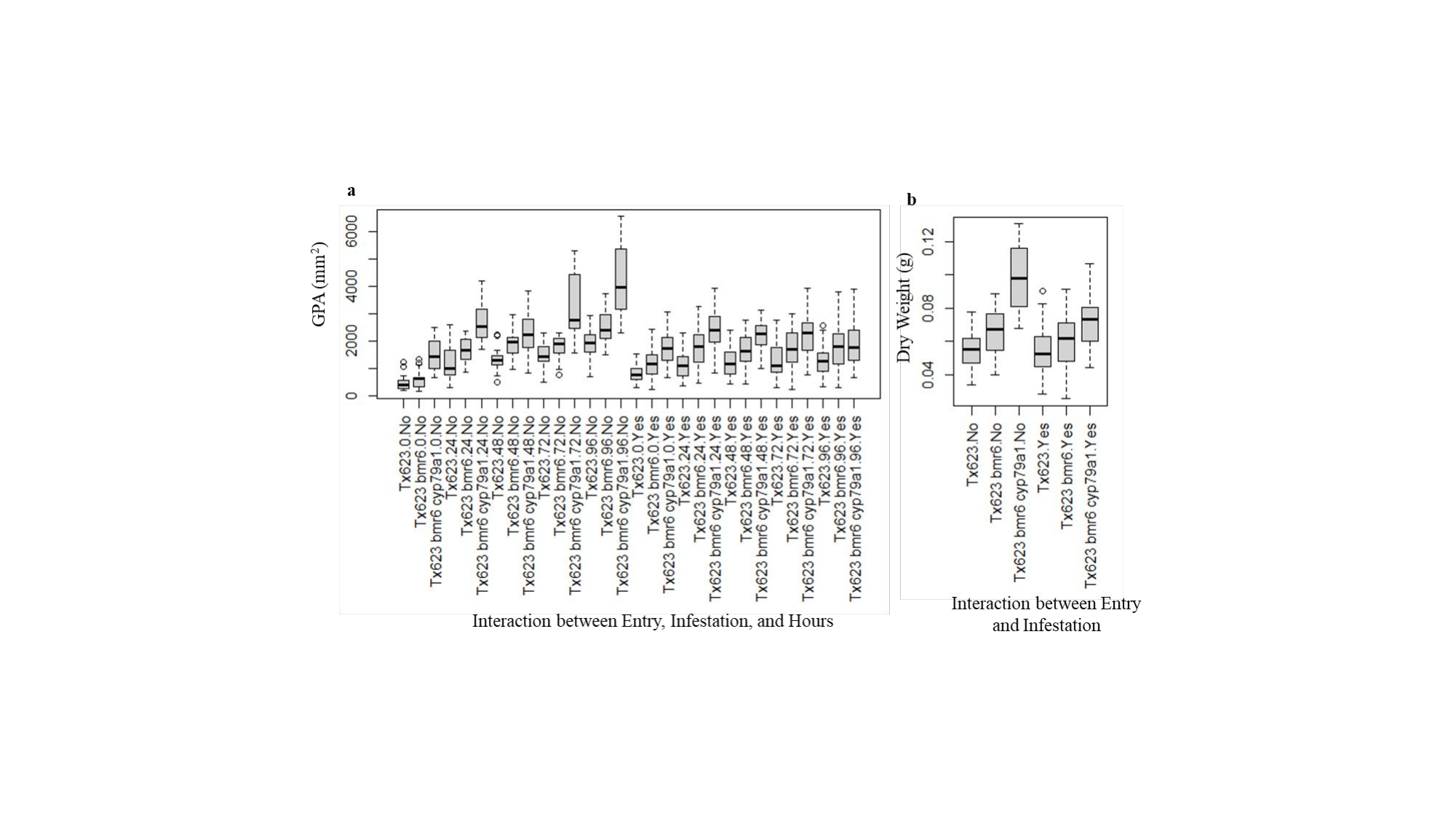

Supplement: Supplementary file 1 — Figure S1. Boxplots showing distributions of the greenhouse data and outliers for (a) ARIS GPA and (b) dry weight. (PPTX 289 KB) [file 122_2021_4017_MOESM1_ESM.pptx]

## Slide 1
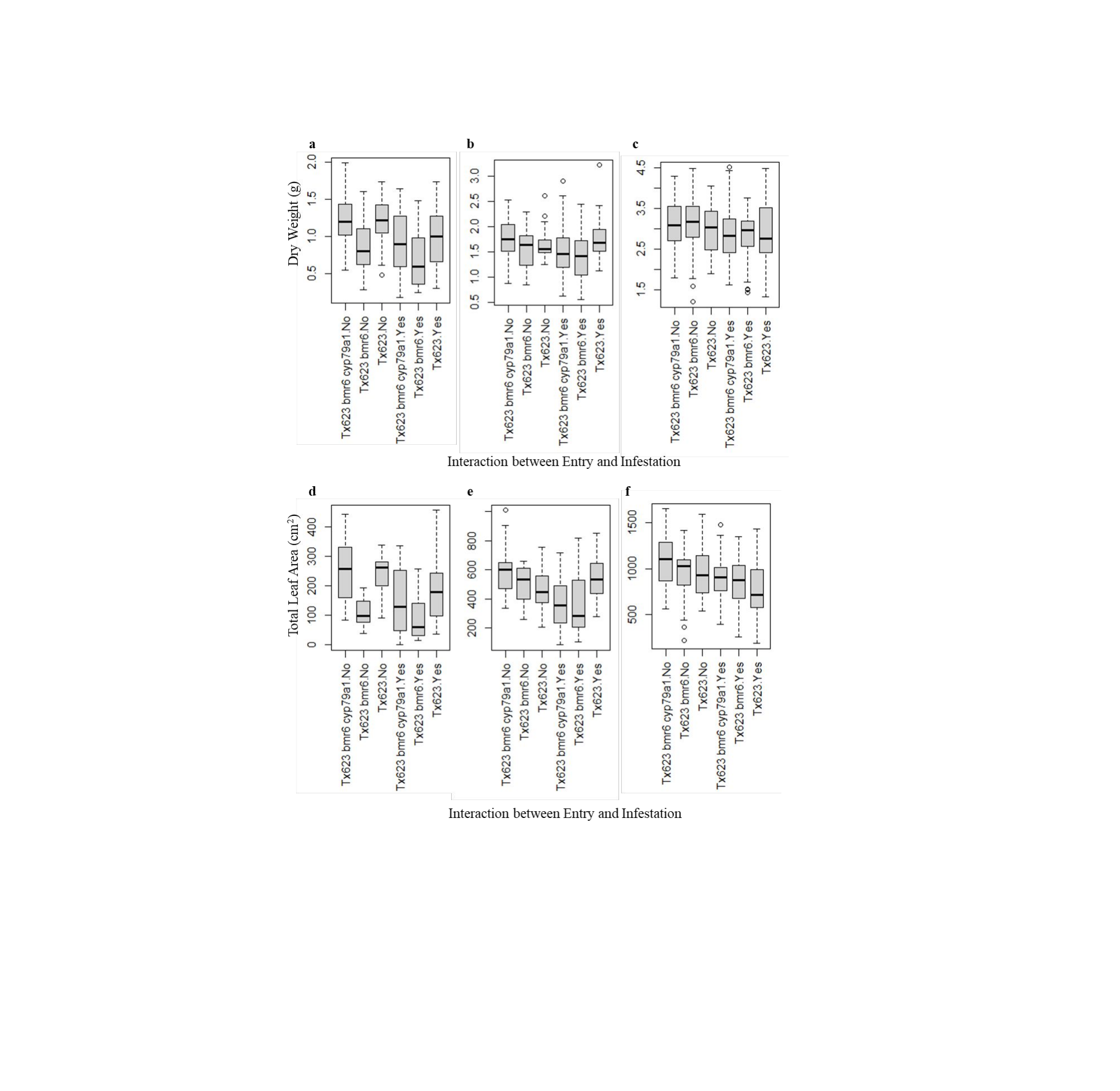

Supplement: Supplementary file 2 — Figure S2. Boxplots showing the distributions of the field data and outliers for (a-c) dry weight and (d-f) total leaf area. (PPTX 314 KB) [file 122_2021_4017_MOESM2_ESM.pptx]
